# Light Illuminated α−Fe_2_O_3_/Pt Nanoparticles as Water Activation Agent for Photoelectrochemical Water Splitting

**DOI:** 10.1038/srep09130

**Published:** 2015-03-16

**Authors:** Xiaodong Li, Zhi Wang, Zemin Zhang, Lulu Chen, Jianli Cheng, Wei Ni, Bin Wang, Erqing Xie

**Affiliations:** 1 Institute of Chemical Materials, China Academy of Engineering Physics, Mianyang 621900, Sichuan, P.R. China; 2 Sichuan Research Center of New Materials, Mianyang 621900, Sichuan, P.R. China; 3 School of Physical Science and Technology, Lanzhou University, Lanzhou 730000, Gansu, P.R. China

## Abstract

The photoelectrochemical (PEC) water splitting is hampered by strong bonds of H_2_O molecules and low ionic conductivity of pure water. The photocatalysts dispersed in pure water can serve as a water activation agent, which provides an alternative pathway to overcome such limitations. Here we report that the light illuminated α−Fe_2_O_3_/Pt nanoparticles may produce a reservoir of reactive intermediates including H_2_O_2_, ·OH, OH^−^ and H^+^ capable of promoting the pure water reduction/oxidation half−reactions at cathode and highly photocatalytic−active TiO_2_/In_2_S_3_/AgInS_2_ photoanode, respectively. Remarkable photocurrent enhancement has been obtained with α−Fe_2_O_3_/Pt as water activation agent. The use of α−Fe_2_O_3_/Pt to promote the reactivity of pure water represents a new paradigm for reproducible hydrogen fuel provision by PEC water splitting, allowing efficient splitting of pure water without adding of corrosive chemicals or sacrificial agent.

The storage of solar energy in chemical bond of H_2_ through water splitting under sun−light presents the most promising strategies to develop a solar−based energetic model in view of the abundant and renewable nature of solar and water resources^1^,^2^,^3^,^4^,^5^. Since the pioneering studies of Fushijima and Honda in the early 1970s^6^, which demonstrated oxidation of water on n−type TiO_2_ single−crystal electrode by band−gap excitation, photoelectrochemical (PEC) water splitting is regarded as the simplest solar to hydrogen (STH) conversion scheme^7^,^8^,^9^,^10^. In a typical PEC water splitting reaction, oxygen is produced on light−excited semiconductor electrode via water oxidation half−reaction 2H_2_O + 4h^+^(hole) → O_2_ + 4H^+^, and hydrogen is generated on Pt counter electrode by water reduction half−reaction 2H_2_O + 2e^−^ → 2OH^−^ + H_2_. Thus, sun light plus water gives us clean hydrogen plus oxygen. It sounds good, but it is not all that easy because the water splitting reaction is an uphill reaction in which the Gibbs free energy increases by 237 kJ mol^−1^^11^. Particularly, splitting of pure water is extremely difficult due to its prohibitively low ionic conductivity. A great deal of effort has been put to overcome the difficulty of splitting of the pure water. Electron donors (sacrificial reagents), including organic compounds (hydrocarbons)^12^,^13^, weak acids^14^,^15^, inorganic ions^16^,^17^,^18^,^19^, etc., are widely used for photocatalytic hydrogen production as they enhances the photocatalytic electron/hole separation by scavenging the photo−generated valence band (VB) holes^20^, resulting in higher quantum efficiency. However, since the electron donors are consumed in this photo−catalytic reaction, the product is only hydrogen and the reaction is not an overall splitting of water. Another way to increase the reactivity of the water splitting is to use the alkaline solutions, which enhance the forward photo−catalytic reaction and suppress backward reaction (recombination of hydrogen and oxygen into water) by scavenging of the photo−generated holes^20^,^21^. It is demonstrated that both of the hydrogen and oxygen production can be increased. The limits of this strategy are low STH conversion efficiency and performance degradation due to the corrosive environment for the electrodes. Thus, it is highly desirable that the PEC water splitting technique which is aimed at providing a clean and renewable fuel can efficiently split water into hydrogen and oxygen without adding of corrosive chemicals or sacrificial agent.

It is generally accepted that the photo−illuminated photocatalysts provide extremely reactive intermediates in water, such as superoxide anion (O_2_), hydroxyl radicals (·OH), and H_2_O_2_, which can reduce/oxidize the pollutants^22^,^23^,^24^,^25^,^26^. In effect, the reactive intermediates generated from photocatalysts are expected to enhance the PEC water splitting efficiency by promoting the water oxidation/reduction half−reaction at photoanode and cathode, respectively. However, the important and unique role of the light−illuminated photocatalysts in water and their consequent ability to serve as water activation agent by generating reactive intermediates for PEC water splitting has not been considered previously.

Here, a new strategy of water activation by generation of various reactive intermediates using the photo−illuminated α−Fe_2_O_3_/Pt nanoparticles (NPs) has been demonstrated for PEC water splitting. α−Fe_2_O_3_ was chosen in our experiment because it has a band−gap of 2.0 to 2.2 eV corresponding to the absorption of 564 to 620 nm light, allowing it a promising photocatalyst for harvesting solar energy for hydrogen production^27^,^28^ or degradation of organic pollutants and toxics^29^,^30^. Furthermore, because the conduction band (CB) bottom (*E*_CB_) and VB top (*E*_VB_) of α−Fe_2_O_3_ is more positive than the hydrogen and oxygen evolution potential, respectively, only reactive intermediates can be generated into water, rather than evolution of hydrogen through photocatalytic water splitting. The application of α−Fe_2_O_3_ as a potential photocatalysts is mainly limited by its short lifetime of photogenerated charge carriers (<10 ps) and short hole diffusion length (~2 to 4 nm)^27^,^31^. To address these issues, Pt NPs were decorated on α−Fe_2_O_3_ by a polyol reduction method, which can serve as cocatalysts to enhance the photocatalytic activity and increase the lifetime of the photogenerated charge carriers^32^,^33^. Combined with the high photocatalytic active TiO_2_/In_2_S_3_/AgInS_2_ photoanode, remarkable photocurrent of ~0.788 mA cm^−2^ at 1.5 V vs. Ag/AgCl has been obtained with α−Fe_2_O_3_/Pt as water activation agent, more than ten times as large as the values without α−Fe_2_O_3_/Pt (0.075 mA cm^−2^ at 1.5 V vs. Ag/AgCl).

## Results

**Figure 1:**
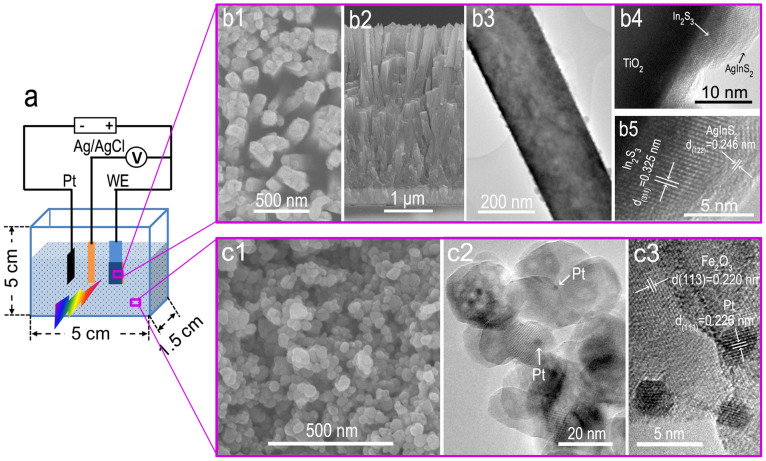
Schematic diagram and morphology and structure characterization. (a) Schematic diagram of PEC cell. (b) FE−SEM (b1 and b2) and TEM (b3–b5) images of TiO_2_/In_2_S_3_/AgInS_2_ electrode. (c) FE−SEM (c1) and TEM (c2 and c3) images of α−Fe_2_O_3_/Pt NPs.

Figure 1a shows a schematic diagram of the reaction vessels, where TiO_2_/In_2_S_3_/AgInS_2_ photoanode pressed on the inner wall of the quartz vessel with conducting side facing the reaction solution serves as the working electrode, Pt−foil as the counter electrode, and Ag/AgCl in saturated KCl as the reference electrode. The photoanode is the central to the PEC cell, whose material and structure both play critical roles in the device performance. An ideal photoanode requires fast water oxidation kinetics at the semiconductor/water interface, fast electron transport and suitable band gap large enough (>1.6 eV) to split water and small enough (<2.2 eV) to absorb a wide range of the solar spectrum. TiO_2_/In_2_S_3_/AgInS_2_ core−shell structure is one of the high−performance photoanodes that satisfy these requirements simultaneously, which is crucial to evaluate the ability of our strategy to promote the water splitting efficiency. Figure 1(b1) and (b2) shows the top and cross-sectional view of the TiO_2_/In_2_S_3_/AgInS_2_ photoanode, respectively. After growth of In_2_S_3_ and AgInS_2_, the products inherit the morphology of the TiO_2_ NW arrays, showing an average diameter of ~116 nm and a length of ~3.36 μm. A typical transmission electron microscopy (TEM) image of a single TiO_2_/In_2_S_3_/AgInS_2_ NW demonstrates that the surface of the TiO_2_ NWs appears to be very coarse, and many NPs are coated over the surface of TiO_2_ NWs, as illustrated in Figure 1(b3). Further insight into the structural information was obtained by high resolution TEM (HRTEM) taken from the TiO_2_/In_2_S_3_/AgInS_2_ interface, Figure 1(b4) and (b5). The In_2_S_3_ buffer layer and TiO_2_ NW form a core−shell structure and the AgInS_2_ NPs decorated on surface of In_2_S_3_. The resolved spacings between the two parallel neighboring fringes are 0.325 and 0.246 nm, corresponding to the [311] plane of cubic In_2_S_3_ and [122] plane of orthorhombic AgInS_2_. The crystal phase of the three materials was further investigated by XRD spectrum; the diffraction peaks in Figure S1 are well indexed with the rutile TiO_2_, cubic In_2_S_3_ and orthorhombic AgInS_2_, respectively.

**Figure 2:**
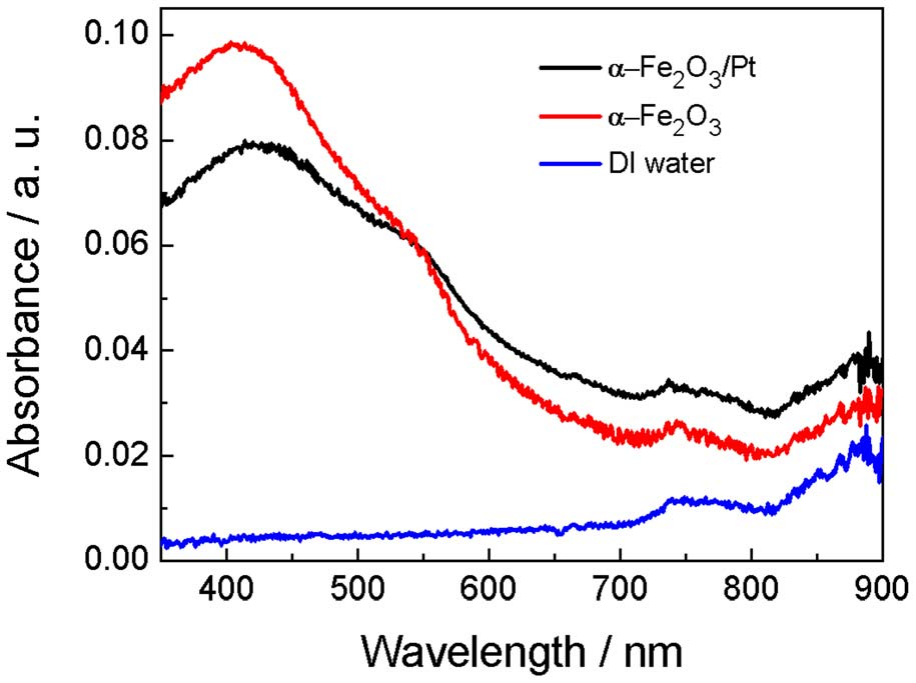
UV–VIS optical absorption spectra of DI water, 0.1 mg/mL α−Fe_2_O_3_ in DI water and 0.1 mg/mL α−Fe_2_O_3_/Pt NPs in DI water.

Pt NPs were deposited on α−Fe_2_O_3_ NPs by the polyol reduction method (see Experimental section and also Ref. 32). As illustrated in Figure 1(c1), the average size of the hybrid α−Fe_2_O_3_/Pt NPs was ~38 nm. Pt NPs with average size of ~3 nm were decorated on α−Fe_2_O_3_ NPs, as confirmed by the TEM image in Figure 1(c2) and (c3). The measured lattice spacing for Pt NPs was 0.226 nm, in agreement with the value for the [111] planes of Pt (JCPDS no. 04−0802). The presence of Pt on α−Fe_2_O_3_ NPs can be further demonstrated by EDS mapping, where Fe, O and Pt elements are distributed uniformly in the hybrid α−Fe_2_O_3_/Pt NPs (see supporting information, Figure S2). Figure 2 compares the UV–vis absorption spectra of α−Fe_2_O_3_/Pt and α−Fe_2_O_3_ NPs dispersed in DI water with that of DI water. The DI water exhibits an intrinsic absorption of water with weak absorption intensity in the visible range; while a broad absorption with the absorption edge at about 600 nm is found for both α−Fe_2_O_3_/Pt and α−Fe_2_O_3_ NPs dispersed in DI water, which originates from the intrinsic absorption of α−Fe_2_O_3_.

**Figure 3:**
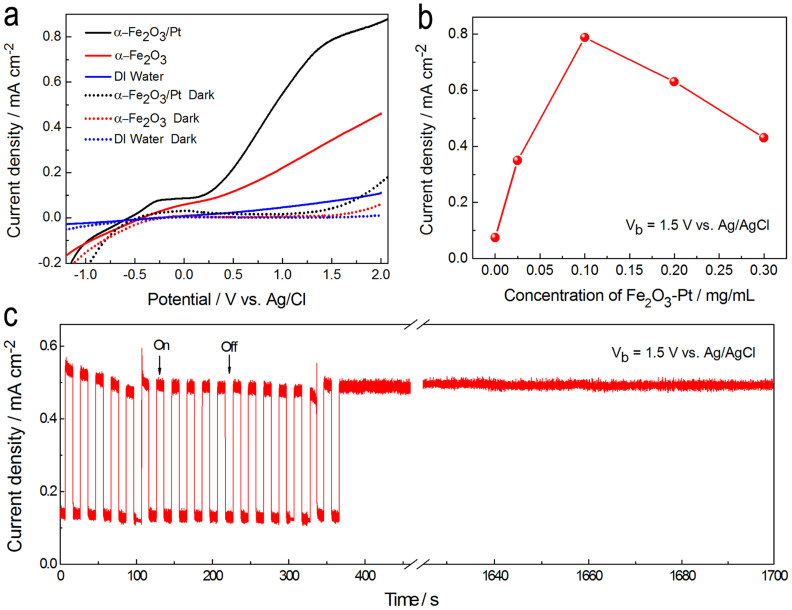
Photoelectrochemical properties. (a) *J*–*V* curves of TiO_2_/In_2_S_3_/AgInS_2_ in different electrolytes of 15 MΩ DI water, 15 MΩ DI water with α−Fe_2_O_3_/NPs and 15 MΩ DI water with α−Fe_2_O_3_/Pt NPs in the dark and under illumination. (b) α−Fe_2_O_3_/Pt concentration−dependent behavior of current density at *V*_b_ = 1.5 V vs. Ag/AgCl. (c) *J*–*t* curve of TiO_2_/In_2_S_3_/AgInS_2_ in DI water containing 0.1 mg/mL α−Fe_2_O_3_/Pt under chopped illumination at a bias of 1.5 V vs. Ag/AgCl. The inset in (c) shows the evolution of H_2_ during the *J*–*t* curve measurement.

To test the ability of the reactive intermediates produced from light illuminated α−Fe_2_O_3_/Pt NPs to enhance the performance of PEC water splitting, we measured the current density versus applied voltage (*J*–*V*) curves of the TiO_2_/In_2_S_3_/AgInS_2_ working electrode in different electrolytes of 15 MΩ DI water, 15 MΩ DI water with α−Fe_2_O_3_/NPs and 15 MΩ DI water with α−Fe_2_O_3_/Pt NPs in the dark and under illumination. The photocurrent in DI water is only 0.075 mA cm^−2^ at 1.5 V vs. Ag/AgCl, which increases sharply to 0.345 mA cm^−2^ with α−Fe_2_O_3_ NPs added to DI water. When tested α−Fe_2_O_3_/Pt as a water activation agent, as expected, the *J* improvement is more pronounced for α−Fe_2_O_3_/Pt than for α−Fe_2_O_3_, to ~0.788 mA cm^−2^ at 1.5 V vs. Ag/AgCl, as shown in Figure 3a. What is more, the *J* value shows a α−Fe_2_O_3_/Pt concentration−dependent behavior, which increases with increasing of the concentration of α−Fe_2_O_3_/Pt, reaching a maximum value at 0.1 mg/mL, followed by decreasing with further increasing concentration, as shown in Figure 3b. Excessive α−Fe_2_O_3_/Pt in water decreases the light penetration depth, which reduces the rate of photo−catalyzed reaction of water and consequent generation of reactive intermediates by α−Fe_2_O_3_/Pt, resulting in the decrease of *J*. It is noteworthy that a minute amount of α−Fe_2_O_3_/Pt NPs were remained in the photoelectrode (Figure S3) after PEC measurement, indicating that the α−Fe_2_O_3_/Pt NPs adsorbed on photoelectrode during the PEC measurement can be neglected.

To compare the effect of the addition of α−Fe_2_O_3_/Pt on the pure water splitting reaction rate to that of the addition of generally used chemicals, two control experiments were conducted by replacing the α−Fe_2_O_3_/Pt suspension with 1 M NaOH aqueous solution and 0.5 M K_2_SO_4_ aqueous solution containing H_2_SO_4_ (adjust the pH to 1.7). As shown in Figure S4, the *J* value is 1.07 mA cm^−2^ at 0.9 V vs. Ag/AgCl for NaOH electrolyte and 0.981 mA cm^−2^ at 1.5 V vs. Ag/AgCl for K_2_SO_4_ electrolyte, which are slightly larger than that of α−Fe_2_O_3_/Pt suspension electrolyte. This result indicates that our strategy of using photocatalysts to promote the reactivity of pure water provide a promising approach for high efficiency PEC water splitting, and the pure water splitting performance could be greatly improved by using more promising semiconducting materials with novel nanostructures in the follow-up works.

Figure 3c shows a representative *J*–*t* curve of TiO_2_/In_2_S_3_/AgInS_2_ in DI water containing 0.1 mg/mL α−Fe_2_O_3_/Pt. The measurement was conducted under the illumination of simulated solar light (AM 1.5 G, 100 mW cm^−2^) at 1.5 V vs. Ag/AgCl. Prior to the measurement, the newly synthesized TiO_2_/In_2_S_3_/AgInS_2_ were illuminated under simulated solar light for 200 s, allowing stabilization of the performance of TiO_2_/In_2_S_3_/AgInS_2_ electrode. From the result one can see that the instantaneous photocurrent density with turning the light on reaches the constant photocurrent density and remains constant until the light is turned off, where the current immediately decays to the dark value of the current. This reproducible rapid rise and decay behavior implies the fast hole scavenging from the surface of the In_2_S_3_/AgInS_2_ heterostructure to the solution and rapid transferring of photoelectrons from In_2_S_3_/AgInS_2_ to current collector via the interior TiO_2_ NWs^34^,^35^. Additionally, the photocurrent was steady for 1700 s, indicating stable photo−stability of both Fe_2_O_3_/Pt and TiO_2_/In_2_S_3_/AgInS_2_ photoanode.

**Figure 4:**
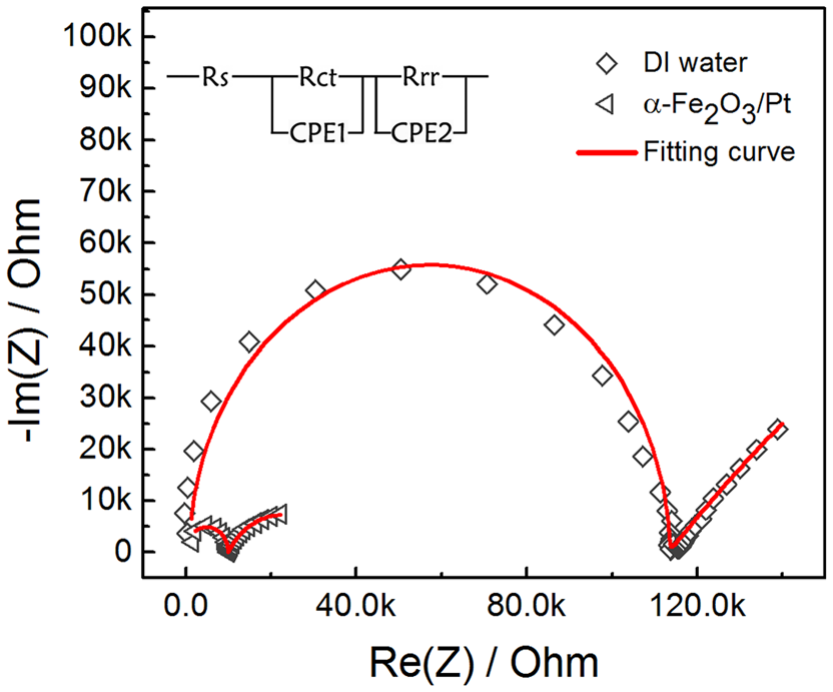
Nyquist plots of TiO_2_/In_2_S_3_/AgInS_2_ in DI water and DI water containing 0.1 mg/mL α−Fe_2_O_3_/Pt measured at open-circuit conditions under simulated solar-light illumination.

To reveal the differences in the interfacial charge-transfer characteristics of both half reactions in the PEC cell with and without α−Fe_2_O_3_/Pt, electrochemical impedance spectroscopy (EIS) measurements were carried out in a two electrode configuration PEC cell^36^. The Nyquist plots of the obtained EIS data measured at open-circuit conditions under simulated solar-light illumination are shown in Figure 4. According to recent analysis on the EIS spectra of the PEC cell for water splitting^36^,^37^, the first semicircle in the high−frequency region (>10^3^ Hz) represents the charge transfer (*R*_ct_) at the TiO_2_/In_2_S_3_/AgInS_2_/electrolyte interface; and the other arc in a frequency range of 100 mHz–10^3^ Hz corresponds to the reduction reaction (*R*_rr_) at the Pt counter electrode. The fitting curves fitted by EIS Spectrum Analyser software using an equivalent circuit shown in the inset match well with the measured EIS data. The fitted *R*_ct_ and *R*_rr_ values for the cells with DI water as electrolyte is as large as 112 kΩ and 527 kΩ, respectively; while *R*_ct_ and *R*_rr_ for the cells with α−Fe_2_O_3_/Pt as water activation agent decreased to 0.985 kΩ and 2.5 kΩ, respectively. The EIS analysis revealed that the presence of α−Fe_2_O_3_/Pt in DI water can greatly promote the activity of water reduction/oxidation half−reactions at counter electrode and TiO_2_/In_2_S_3_/AgInS_2_ photoanode, respectively.

## Discussion

**Figure 5:**
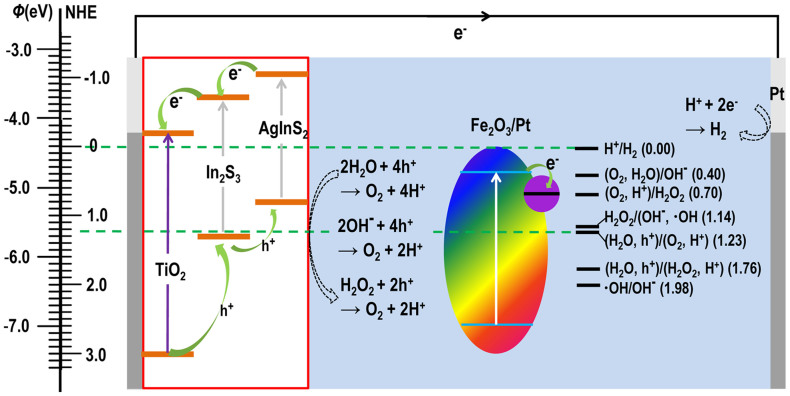
Energetics of operation of the PEC cell with light illuminated α−Fe_2_O_3_/Pt NPs as water activation agent. Potentials for the possible reactions that can be initiated by electron−hole pairs generated in α−Fe_2_O_3_/Pt NPs are standard *E*^0^ values.

On the basis of the above experiments, it is reasonable to ascribe the significant improvement of the PEC water splitting efficiency to the generation of the reactive intermediates from the light illuminated α−Fe_2_O_3_/Pt NPs. Some potential reactions that could be initiated by photo electron−hole pairs generated in α−Fe_2_O_3_/Pt and the consequential process in relation to the water splitting can be depicted as in Figure 5. Under light illumination, the electrons in the VB of α−Fe_2_O_3_ are promoted to the CB of α−Fe_2_O_3_ by photo excitation (γ), and electron (e^−^) − hole (h^+^) pairs are generated. The *E*_CB_ of α−Fe_2_O_3_ (0.38 V vs. NHE) is more negative than the reduction potential to form OH^−^ (0.40 V vs. NHE^38^) and H_2_O_2_ (0.70 V vs. NHE^38^), allowing generation of OH^−^ and H_2_O_2_ through O_2_ + 2H_2_O + 4e^−^ → 4OH^−^ and O_2_ + 2H^+^ + 2e^−^ → H_2_O_2_, respectively. Previous study has predicted that H_2_O_2_ can also be produced via the oxidation of water in the absence of added electron donors via 2H_2_O + 2h^+^ → H_2_O_2_ + 2H^+39^. In a PEC cell, the H_2_O_2_ can be directly oxidized to O_2_ by the photogenerated holes in photoanode through H_2_O_2_ + 2h^+^ → O_2_ + 2H^+^ or reduced to ·OH and OH^−^ by the conduction band electron through H_2_O_2_ + e^−^ → ·OH + OH^−^39,40; and the resultant ·OH may be further reduced to OH^−^ through ·OH + e^−^ → OH^−^38. The resulting H_2_O_2_, OH^−^ and H^+^ in the above reactions are highly active for the water reduction/oxidation half−reaction at the cathode and photoanode in a PEC cell. Thus, the α−Fe_2_O_3_/Pt dispersed in pure water can serve as a water activation agent under light illumination, which could produce a reservoir of reactive intermediates (H_2_O_2_, ·OH, OH^−^, H^+^) capable of promoting the water splitting reaction.

**Figure 6:**
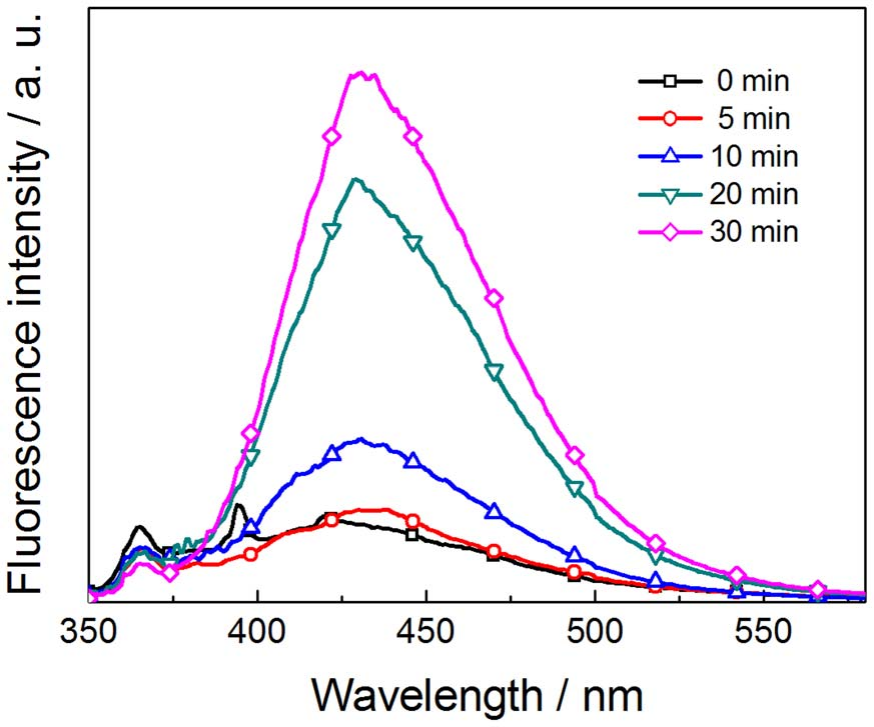
Fluorescence spectral changes recorded during illumination of α−Fe_2_O_3_/Pt suspension containing 0.5 mM terephthalic acid at various irradiation periods.

According to the above mentioned reaction mechanism, ·OH is a key intermediate relating to generation of substances (H_2_O_2_, OH^−^ and H^+^) that can directly promote the water splitting reaction at photoanode and counter electrode. It is widely accepted that the fluorescent probe method using terephthalic acid (TA) as the ·OH capture is a highly sensitive technique, in which the TA reacts with ·OH and generates luminescent 2-hydroxyterephthalic acid (TAOH) with a characteristic peak at ~426 nm^41^,^42^,^43^. Figure 6 shows the fluorescence spectral changes observed during illumination of α−Fe_2_O_3_/Pt suspension containing 0.5 mM terephthalic acid at various irradiation periods. Gradual increase in the fluorescence intensity at ~428 nm with increasing illumination time implies that fluorescent TAOH was formed via the specific reaction between ·OH and TA during illumination of α−Fe_2_O_3_/Pt suspension, which is a direct evidence of the presence of ·OH. In addition, H_2_O_2_ is another important intermediate, the existence of which can be verified by hydrogen peroxide indicator strip, as illustrated in Supporting Information, Figure S5. The observation of ·OH and H_2_O_2_ in the water splitting reaction provide a weighty evidence to the reaction mechanism.

**Figure 7:**
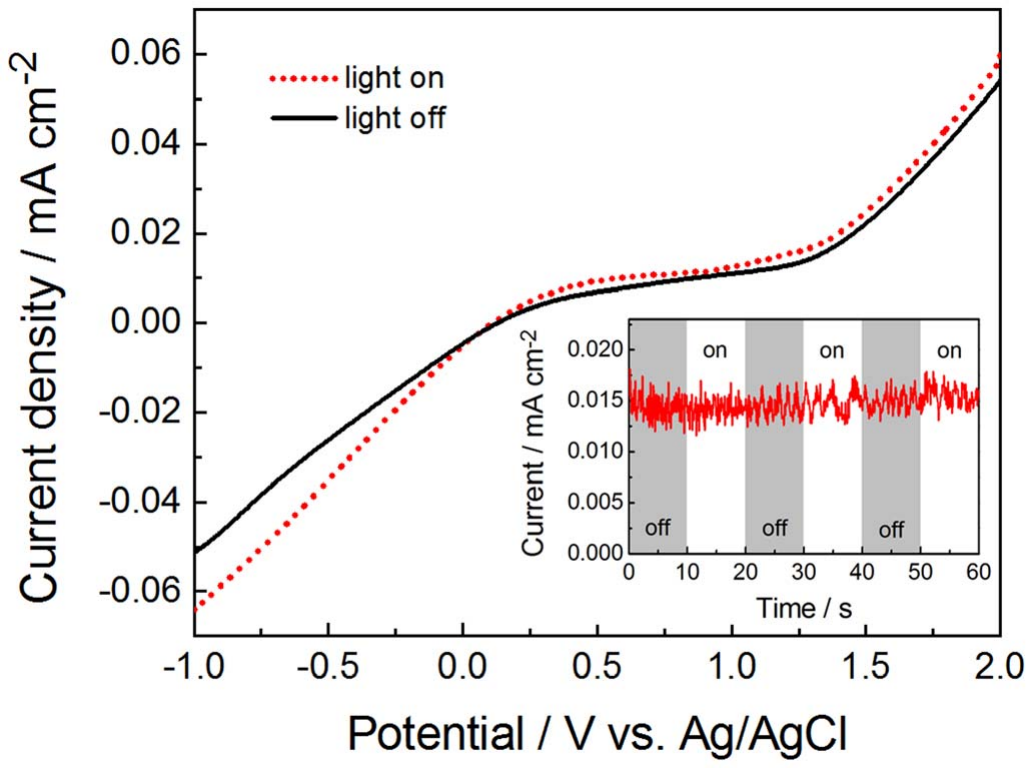
*J*–*V* curves of a PEC cell with a Pt−foil as working electrode, another Pt−foil as counter electrode and α−Fe_2_O_3_/Pt NPs dispersed in DI water as electrolyte in dark and under light illumination. The inset shows the *J*–*t* curve of the PEC cell under chopped illumination.

Because the *E*_CB_ of α−Fe_2_O_3_ (0.38 V vs. NHE) is less negative than the hydrogen evolution potential (0.00 V vs. NHE), it is not able to reduce the H^+^ to give H_2_ directly by α−Fe_2_O_3_. Therefore, in the PEC cell with α−Fe_2_O_3_/Pt activated pure water as reaction solution, the reduction of H^+^ takes place only on Pt cathode, which requires efficient oxidation of the water or reactive intermediates including H_2_O_2_, OH^−^ by the photoanode. Thus the water oxidation ability of the photoanode plays a key role for water splitting in this PEC cell. In this work, we investigated three TiO_2_ NW based electrodes including TiO_2_ NW, TiO_2_ NW/CdS and TiO_2_/In_2_S_3_/AgInS_2_. Identical behavior has been observed for the three photoanodes, as illustrated in Figure 3, Figure S6 and S7. Due to the excellent light harvesting (see Supporting Information, Figure S8) and photocatalytic activity property^44^, the TiO_2_/In_2_S_3_/AgInS_2_ working electrode shows the highest photocurrent value. To double check the reasonability of this conclusion and the reaction mechanism described in Figure 5, we compare the *J*–*V* curves of a PEC cell with a Pt−foil as working electrode, another Pt−foil as cathodic electrode and α−Fe_2_O_3_/Pt NPs dispersed in DI water as electrolyte in dark and under light illumination. As shown in Figure 7, identical behavior has been observed for *J*–*V* curves with and without light illumination. Furthermore, no instantaneous photocurrent was observed on the chopped−light current density versus time (*J*–*t*) curve of the cell. These results indicate that oxidation of water at the working electrode (Figure 5) plays a key role for water splitting in our PEC cell.

In conclusion, an alternative pathway to activate the pure water for PEC water splitting by introducing photocatalysts into water has been developed. The light illuminated α−Fe_2_O_3_/Pt NPs may produce a reservoir of reactive intermediates including H_2_O_2_, ·OH, OH^−^ and H^+^ capable of promoting the water reduction/oxidation half−reactions at cathode and TiO_2_/In_2_S_3_/AgInS_2_ photoanode, respectively. Remarkable photocurrents of ~0.788 mA cm^−2^ at 1.5 V vs. Ag/AgCl has been obtained with α−Fe_2_O_3_/Pt as water activation agent, more than ten time as large as the values without α−Fe_2_O_3_/Pt (0.075 mA cm^−2^ at 1.5 V vs. Ag/AgCl). The present results provide a fertile base for further investigation. The strategy of using photocatalysts to generate reactive intermediates in pure water for PEC water splitting demonstrated by α−Fe_2_O_3_/Pt NPs can be leveraged to other, more promising semiconducting materials with novel nanostructures to greatly improve their efficiencies and application areas. The approach could also be extended to other energy and artificial photosynthesis applications.

## Methods

### Preparation of α−Fe_2_O_3_/Pt NPs

α−Fe_2_O_3_ NPs with diameter ~30 nm were purchased from Aladdin Industrial Inc. (Shanghai, China). Deposition of Pt onto α−Fe_2_O_3_ NPs followed procedures outlined previously^32^. Typically, α−Fe_2_O_3_ powder (0.5 g) was dispersed in a mixed solution (40 mL) containing H_2_PtCl_6_ aqueous solution (1 wt.%) and ethanol under ultrasonication for 30 min. Then the slurry was dried at 60°C. Ethylene glycol (40 mL) was added to the dry powder followed by stirring and ultrasonication to form a homogenous suspension. The suspension was kept at 100°C in dark for 6 h. At last the α−Fe_2_O_3_/Pt powder was collected by centrifugation, washed with distilled water for several times, dried at 60°C and sintered at 400°C for 20 min.

### Preparation of TiO_2_/In_2_S_3_/AgInS_2_ core−shell electrodes

At first, a TiO_2_ polymeric sol was prepared by the sol gel process according to the previous reports^45^. Then the TiO_2_ sol was spin−coated on the fluorine−doped SnO_2_ (FTO) substrates followed by annealing at 450°C for 2 h. The TiO_2_ NW arrays were grown directly on seeded FTO substrates by using the hydrothermal method reported previously^46^. In a typical synthesis process, titanium (IV) butoxide (0.5 g) was added into an aqueous HCl solution (25 mL of deionized water and 25 mL of concentrated HCl (38%)) under magnetic stirring. The solution was stirred for another 10 min and then poured into a Teflon−lined stainless steel auto−clave (100 mL capacity). Six pieces of the seeded−FTO (0.8 cm × 2 cm, with seeded area of 0.8 cm^−2^) were placed at an angle against the wall of the Teflon−liner with the conducting side facing down. The autoclave was sealed, heated to 170°C and held at the temperature for 6 h. After cooling down to room temperature, the obtained products were washed successively by DI water and ethanol and finally annealed at 500°C for 2 h.

In_2_S_3_/AgInS_2_ were deposited on TiO_2_ NWs by sequential chemical bath deposition (S-CBD) method according to a previous report but with a modified recipe^44^. Typically, the TiO_2_ NWs on FTO substrate were successively dipped into InCl_3_·4HO_2_ ethanol solution (3 mM) for 4 min, ethanol for 1 min, Na_2_S·9H_2_O water-methanol solution (3 mM) (1:1 volume ratio) for 4 min and water-methanol (1:1 volume ratio) mixture for 1 min at 25°C. The desired deposition of In_2_S_3_ was achieved after 12 cycles with the white TiO_2_ NW film gradually became pale yellow. Subsequently, the TiO_2_/In_2_S_3_ film was immersed in AgNO_3_ ethanol solution (2 mM) at 25°C for 2 min. The resultant brown TiO_2_/In_2_S_3_/AgInS_2_ films were washed with ethanol and sintered at 400°C for 30 min in N_2_ atmosphere.

### Photoelectrochemical measurements

All the PEC measurements were performed in a quartz reaction vessel containing DI water (20 mL, 15.0 MΩ, Elix Advantage 10, Merck Millipore) and α−Fe_2_O_3_/Pt NPs. The PEC measurements were performed in a three electrode configuration with TiO_2_/In_2_S_3_/AgInS_2_ as the working electrode, Pt−foil (surface area of 1.0 cm^2^) as the counter electrode, and Ag/AgCl in saturated KCl as the reference electrode. To prevent suspended α−Fe_2_O_3_/Pt NPs from screening the photo−absorption of the photoelectrode, the TiO_2_/In_2_S_3_/AgInS_2_ electrodes were pressed against the inner wall of the quartz vessel with conducting side facing the reaction solution. The TiO_2_/In_2_S_3_/AgInS_2_ electrode was connected to the measuring instrument by pressing a Pt foil on the FTO layer of the TiO_2_/In_2_S_3_/AgInS_2_ electrode. The PEC performances were measured using an Electrochemical Workstation (Bio–Logic SAS, VSP–300). Illumination was from a solar simulator with a Xe arc lamp as light source and the spectrum was matched to the AM 1.5 G spectrum. Before the measurement, the solar intensity (100 mW cm^−2^) was calibrated with a reference silicon solar cell. The illuminated area of the working electrode was 0.8 cm^2^.

Hydroxyl radical formation was studied by means of terephthalic acid (TA) fluorescence probe method as follows. An aqueous solution containing 0.5 mM TA was prepared, and then α−Fe_2_O_3_/Pt NPs (0.1 mg/mL) was suspended in this solution in a quartz reaction vessel. Prior to irradiation, the suspension was magnetically stirred for 30 min in a dark box to establish an adsorption−desorption equilibrium. The excitation light source was the same as that in PEC water splitting measurements. To sediment α−Fe_2_O_3_/Pt NPs from the suspensions and get rid of light scattering for the subsequent measurement of the fluorescence spectra, the samples for different irradiation periods were centrifuged at 10000 rpm for 2 min. Fluorescence spectra of 2-hydroxyterephthalic acid (TAOH) were measured on a fluorescence spectrophotometer (Omni-pR-PL, Beijing Zolix Instruments CO., LTD) with an excitation at 325 nm light.

### Characterizations

The morphology and microstructure of the TiO_2_/In_2_S_3_/AgInS_2_ electrode and α−Fe_2_O_3_/Pt NPs were characterized by a field emission scanning electron microscopy (FE−SEM, Hitachi S−4800) and transmission electron microscopy (TEM, FEI Tecnai F30). Elemental analysis was performed on an energy−dispersive x−ray (EDX) spectroscopy attached to the FE−SEM. X−Ray diffraction spectra (XRD) was collected on a Bruker D8 Advance X−ray diffractometer using a Cu Kα source (λ = 0.154056 nm). The optical absorbance spectra were acquired using a UV–visible spectrophotometer (TU−1901). Electrochemical impedance spectroscopy (EIS) was measured with the Electrochemical Workstation in a two electrode configuration within a frequency range from 0.1 Hz to 800 kHz at open-circuit voltage with a potential pulse of 100 mV in amplitude under simulated solar-light illumination (AM 1.5 G, 100 mW cm^−2^). Prior to the recording of EIS data, the PEC cell was illuminated for 10 min at an applied bias of 1.5 V to establish equilibrium of the system. The EIS data were fitted by EIS Spectrum Analyser software. Hydrogen peroxide indicator strips (Quantofix Peroxide 25, MACHEREY–NAGEL, Germany) were used to test the existence of H_2_O_2_.

## Supplementary Material

Supplementary Information Supplemental information
