# Supplementary material for: Light Illuminated α−Fe2O3/Pt Nanoparticles as Water Activation Agent for Photoelectrochemical Water Splitting
Source: Sci Rep. 2015 Mar 16;5:9130. doi: 10.1038/srep09130 (PMC5390902; doi:10.1038/srep09130)
Supplement: Supplementary Information — Supplemental information [file srep09130-s1.pdf]

Supplementary information

Light Illuminated $\alpha-Fe_2O_3$/Pt Nanoparticles as Water Activation Agent for Photoelectrochemical Water Splitting

Xiaodong Li,[1,2] Zhi Wang,[1] Zemin Zhang,[3] Lulu Chen,[3] Jianli Cheng,[1,2] Wei Ni,[1,2] Bin Wang,[1,2] Erqing Xie[3]

[1]*Institute of Chemical Materials, China Academy of Engineering Physics, Mianyang 621900, Sichuan, P.R. China.* [2]*Sichuan Research Center of New Materials, Mianyang 621900, Sichuan, P.R. China.* [3]*School of Physical Science and Technology, Lanzhou University, Lanzhou 730000, Gansu, P.R. China.*

* Correspondence and requests for materials should be addressed to X. Li (lixdong10@gmail.com) or B. Wang (edward.bwang@gmail.com).

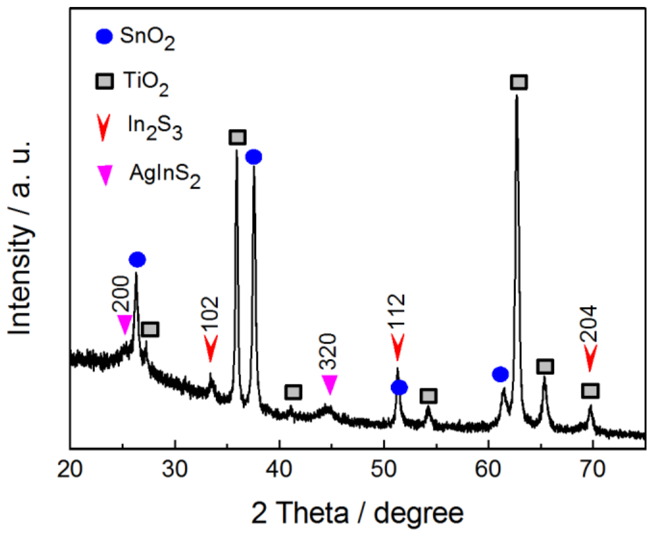

Figure S1 XRD spectrum of $TiO_2/In_2S_3/AgInS_2$.

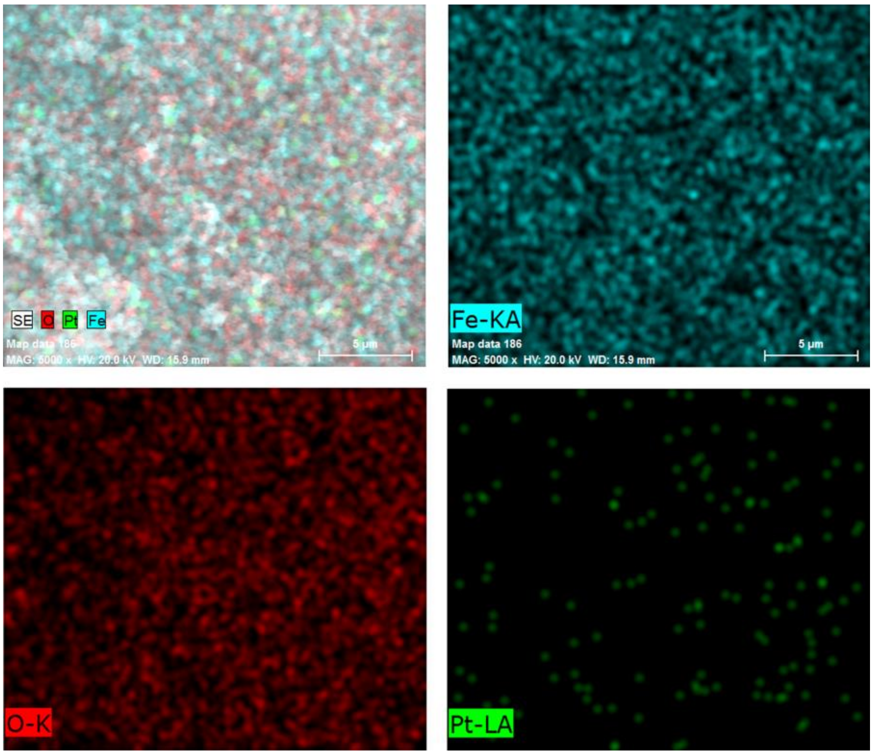

Figure S2 EDS mapping of α−Fe_2O_3/Pt NPs showing uniform distribution of Fe, O and Pt elements in the hybrid α−Fe_2O_3/Pt NPs.

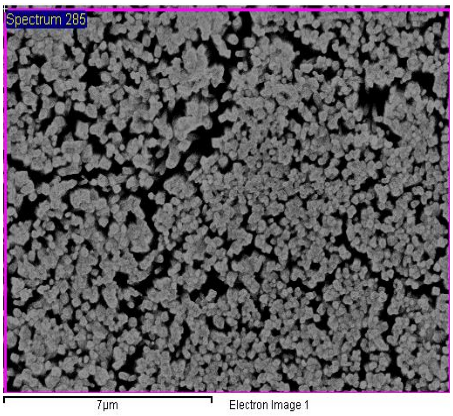

Element	Weight%	Atomic%
O K	7.78	65.11
S K	0.48	1.99
Ti K	11.35	31.73
Fe K	0.05	0.13
Ag L	0.32	0.40
In L	0.55	0.64
Totals	20.52	100

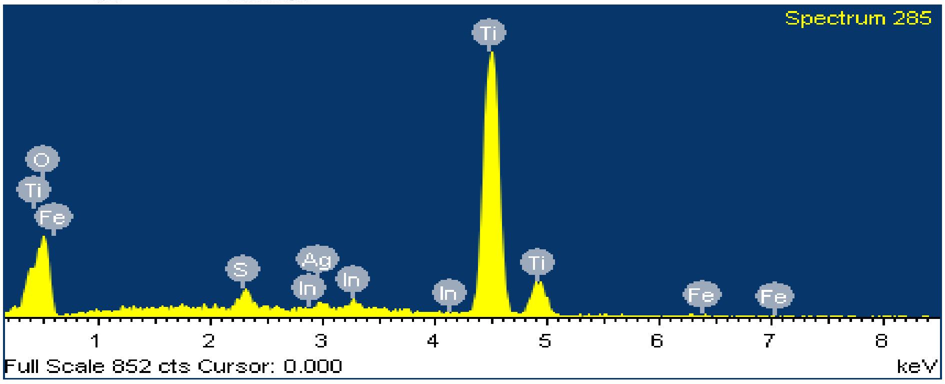

Figure S3 EDX spectrum showing elemental composition of the $TiO_2/In_2S_3/AgInS_2$ electrode after PEC test in DI water containing $\alpha-Fe_2O_3$/Pt NPs. A minute amount of $\alpha-Fe_2O_3$/Pt NPs residue is observed.

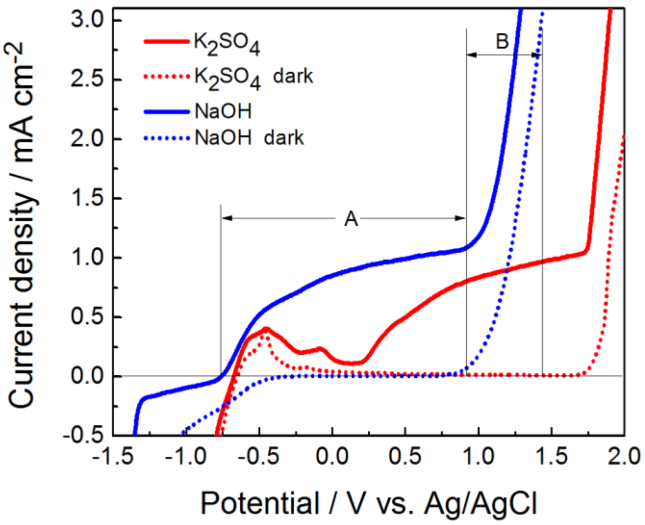

Figure S4 *J*–*V* curves of $TiO_2/In_2S_3/AgInS_2$ in 1 M NaOH aqueous solution and 0.5 M K_2SO_4 aqueous solution containing H_2SO_4 (adjust the pH to 1.7). The current density in Region A result from PEC water splitting and the sudden increase in Region B should be due to the electrolysis of water.

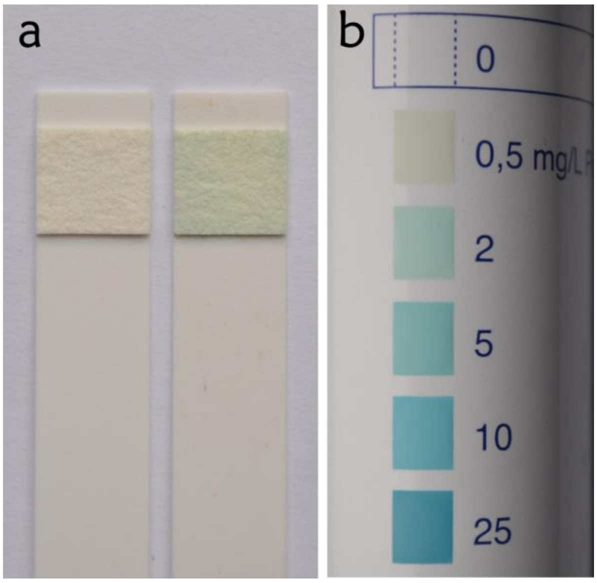

Figure S5 Digital photos showing (a) color changes of the hydrogen peroxide indicator strips dipping into the α–Fe_2O_3/Pt suspension before (Left) and after (Right) 20 min simulated solar light illumination and (b) standard colorimetric card.

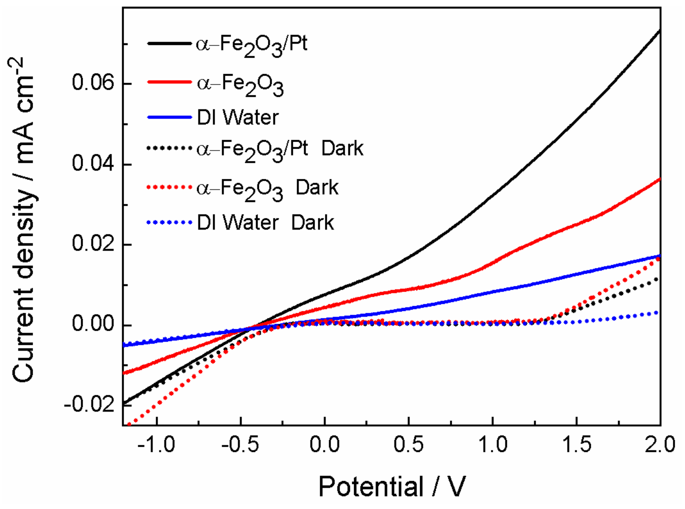

Figure S6 *J*−*V* curves of TiO_2 NWs in different electrolytes of 15 MΩ DI water, 15 MΩ DI water with α−Fe_2O_3/NPs and 15 MΩ DI water with α−Fe_2O_3/Pt NPs in the dark and under illumination.

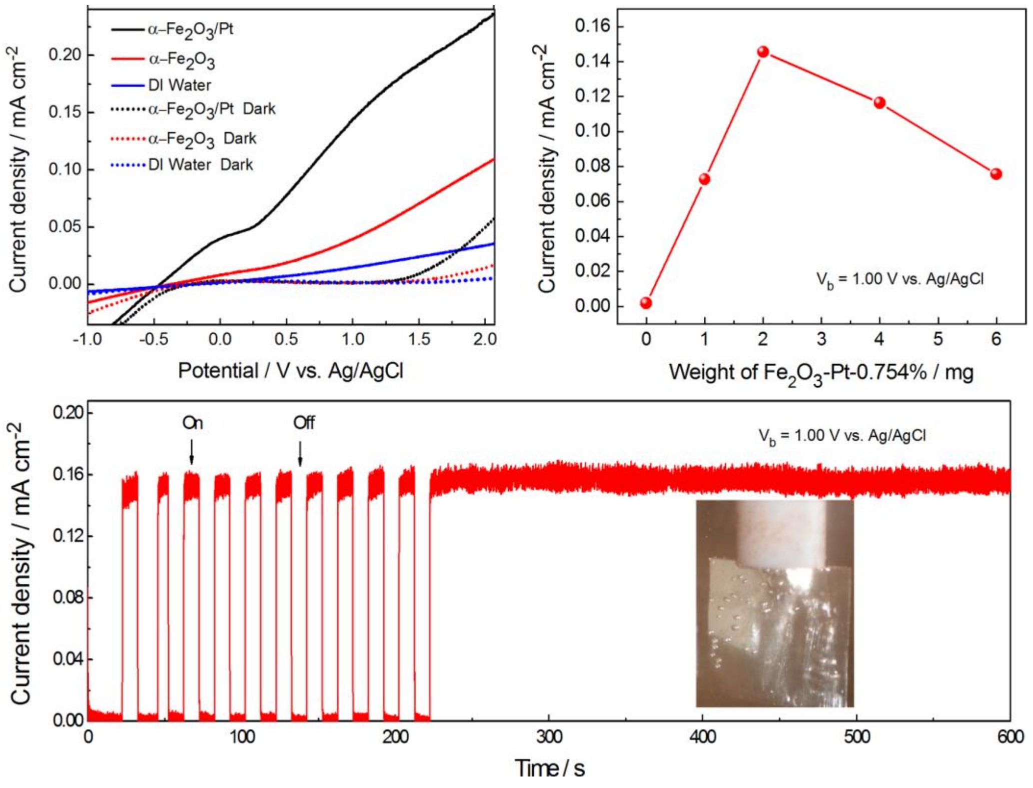

Figure S7 *J*−*V* curves of TiO_2 NW/CdS in different electrolytes of 15 MΩ DI water, 15 MΩ DI water with α−Fe_2O_3/NPs and 15 MΩ DI water with α−Fe_2O_3/Pt NPs in the dark and under illumination.

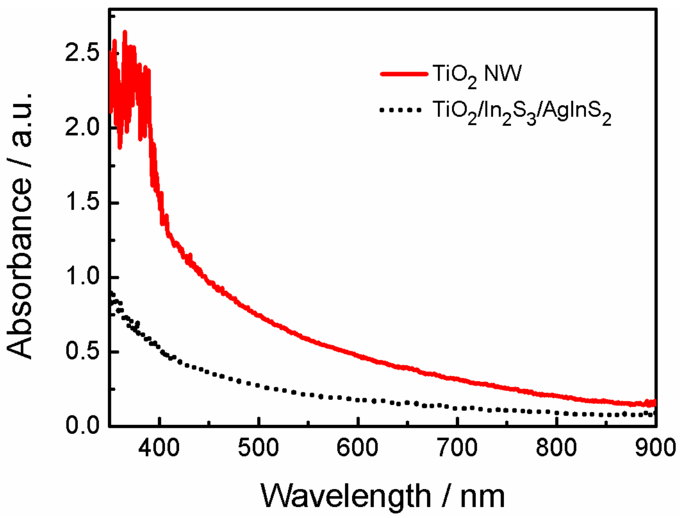

Figure S8 UV–vis optical absorption spectra of TiO_2 NW and $TiO_2/In_2S_3/AgInS_2$ photoanode.
